# *N*-[2-(3-Methyl-1-oxo-1,2-dihydro­pyrrolo­[1,2-*a*]pyrazin-2-yl)eth­yl]methane­sulfonamide

**DOI:** 10.1107/S1600536810026115

**Published:** 2010-07-10

**Authors:** Salman Tariq Khan, Peng Yu, Suchada Chantrapromma, Nighat Afza, Aisha Nelofar

**Affiliations:** a Pharmaceutical Research Centre, PCSIR Labs Complex, Karachi 75280, Pakistan; b Department of Pharmaceutical Engineering, Biotechnology College, Tianjin University of Science & Technology (TUST), Tianjin 300457, People’s Republic of China; c Crystal Materials Research Unit, Department of Chemistry, Faculty of Science, Prince of Songkla University, Hat-Yai, Songkhla 90112, Thailand

## Abstract

In the title compound, C_11_H_15_N_3_O_3_S, the dihedral angle between the five- and six-membered rings is 1.13 (18)°. The ethyl­methane­sulfonamide group is in a (+)synclinal conformation. In the crystal, inter­molecular N—H⋯O and C—H⋯O hydrogen-bond inter­actions link mol­ecules into zigzag ribbons parallel to the *b* axis. The ribbons are further connected by C—H⋯π inter­actions.

## Related literature

For the biological activity of pyrrolo­pyrazinone derivatives, see: Dubis *et al.* (1995 ▶); Micheli *et al.* (2008 ▶); Wang *et al.* (2004 ▶); Zöllinger *et al.* (2007 ▶). For standard bond-length data, see: Allen *et al.* (1987 ▶). For hydrogen-bond motifs, see: Bernstein *et al.* (1995 ▶).
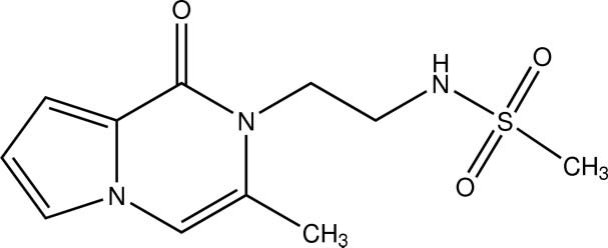


## Experimental

### 

#### Crystal data

C_11_H_15_N_3_O_3_S
*M* *_r_* = 269.33
Monoclinic, 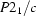

*a* = 5.492 (1) Å
*b* = 20.631 (4) Å
*c* = 11.212 (2) Å
β = 99.953 (6)°
*V* = 1251.3 (4) Å^3^
*Z* = 4
Mo *K*α radiation
μ = 0.26 mm^−1^
*T* = 113 K
0.18 × 0.12 × 0.10 mm

#### Data collection

Rigaku Saturn CCD area-detector diffractometer
Absorption correction: multi-scan (*CrystalClear*; Rigaku, 2005 ▶) *T*_min_ = 0.954, *T*_max_ = 0.974
9267 measured reflections
2969 independent reflections
2499 reflections with *I* > 2σ(*I*)
*R*_int_ = 0.036

#### Refinement

*R*[*F* ^2^ > 2σ(*F* ^2^)] = 0.040
*wR*(*F* ^2^) = 0.105
*S* = 1.05
2969 reflections
169 parameters
H atoms treated by a mixture of independent and constrained refinement
Δρ_max_ = 0.22 e Å^−3^
Δρ_min_ = −0.46 e Å^−3^

### 

Data collection: *CrystalClear* (Rigaku, 2005 ▶); cell refinement: *CrystalClear*; data reduction: *CrystalClear*; program(s) used to solve structure: *SHELXTL* (Sheldrick, 2008 ▶); program(s) used to refine structure: *SHELXTL*; molecular graphics: *SHELXTL*; software used to prepare material for publication: *SHELXTL* and *PLATON* (Spek, 2009 ▶).

## Supplementary Material

Crystal structure: contains datablocks I, global. DOI: 10.1107/S1600536810026115/rz2471sup1.cif

Structure factors: contains datablocks I. DOI: 10.1107/S1600536810026115/rz2471Isup2.hkl

Additional supplementary materials:  crystallographic information; 3D view; checkCIF report

## Figures and Tables

**Table 1:** Hydrogen-bond geometry (Å, °) *Cg*1 is the centroid of the N2/C4–C7 ring.

*D*—H⋯*A*	*D*—H	H⋯*A*	*D*⋯*A*	*D*—H⋯*A*
N3—H1*N*3⋯O1^i^	0.82 (2)	1.99 (2)	2.7923 (18)	168 (2)
C4—H4*A*⋯O3^ii^	0.95	2.36	3.258 (2)	157
C8—H8*A*⋯*Cg*1^iii^	0.99	2.96	3.5153 (19)	116

Symmetry codes: (i) 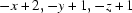; (ii) 
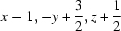
; (iii) 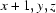.

**Fig. 1.**
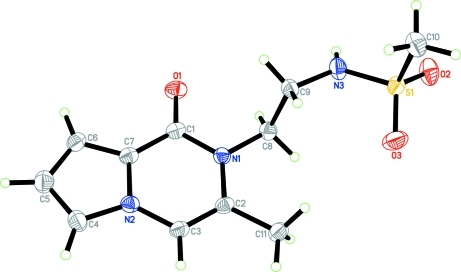
The molecular structure of the title compound, showing 50% probability displacement ellipsoids and the atom-numbering scheme.

**Fig. 2.**
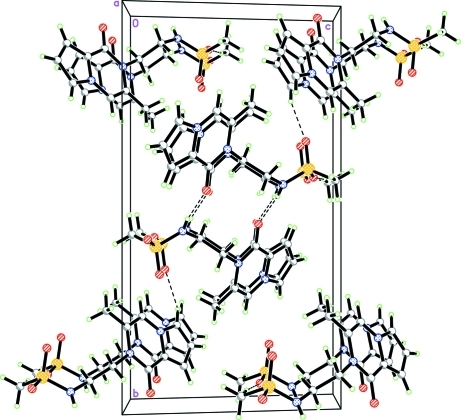
The crystal packing of the title compound viewed along the a axis. Hydrogen bonds are drawn as dashed lines.

## supplementary crystallographic information

### Comment

Pyrrolopyrazinone compounds have been found to possess antitumor activity (Zöllinger *et al.*, 2007), antifeedant effect on storage pests (Dubis *et al.*, 1995) and to be potent and selective non-competitive mGluR5 antagonists (Micheli *et al.*, 2008). Due to the interesting biological activities of pyrrolopyrazinone compounds, the title compound, which may have an improved analgesic activity (Wang *et al.*, 2004), was synthesized and its crystal structure is reported here.

In the title compound (Fig. 1), the nine non-hydrogen atoms of the pyrrolopyrazine ring system are nearly coplanar (*r.m.s.* deviation 0.0107 (2) Å) and the dihedral angle between the five and six membered rings is 1.13 (18)°. The ethylmethanesulfonamide group (C8–C10/N3/S1/O2–O3) is in (+)-synclinal conformation, as indicated by the C1–N1–C8–C9 torsion angle of 83.75 (16)°. The dihedral angle between the mean planes through C8/C9/C10 and N3/C9/S1 is 79.58 (19)°. The bond lengths are in normal ranges (Allen *et al.*, 1997). In the crystal structure (Fig. 2), centrosymmetrically related molecules are linked by N—H···O hydrogen bonds (Table 1) into dimers forming fourteen-membered rings with *R*_2_^2^(14) motifs (Bernstein *et al.*, 1995). Adjacent dimers are linked by C—H···O hydrogen interactions into zigzag ribbons running parallel to the *b* axis. The crystal packing is further stabilized by inter-ribbon C—H···π interactions (Table 1; *Cg*1 is the centroid of the N2/C4–C7 ring).

### Experimental

The title compound was prepared by reacting 2-(2-aminoethyl)-3-hydroxy-3-methyl-3,4-dihydropyrrolo[1,2-*a*]pyrazin-1(2*H*)-one (2.39 mmol) and methanesulfonyl chloride (4.76 mmol) in pyridine (2 ml) for 4 h. The reaction mixture was then poured into ice cold water and the solid obtained was filtered and washed thoroughly with water and then dissolved in aqueous NaHCO_3_ solution. Filtration and then the acidification with dilute HCl gave the title compound as precipitate, which was then filtered and dried. Colourless block-shaped single crystals of the title compound suitable for *X*-ray structure determination were recrystalized from a dichloromethane/methanol solution (9.5:0.5 *v*/*v*) on slow evaporation of the solvent at room temperature after several days.

### Refinement

The amide H atom was located in a difference Fourier map and refined isotropically. The remaining H atoms were placed in calculated positions with d(C—H) = 0.95 Å for aromatic, 0.99 for CH_2_ and 0.98 Å for CH_3_ atoms. The *U*_iso_ values were constrained to be 1.5*U*_eq_ of the carrier atom for methyl H atoms and 1.2*U*_eq_ for the remaining H atoms. A rotating group model was used for the methyl groups. The highest residual electron density peak is located at 0.64 Å from C7 and the deepest hole is located at 0.74 Å from S1.

### Crystal data

**Table:**

C_11_H_15_N_3_O_3_S	*F*(000) = 568
*M**_r_* = 269.33	*D*_x_ = 1.430 Mg m^−^^3^
Monoclinic, *P*2_1_/*c*	Mo *K*α radiation, λ = 0.71073 Å
Hall symbol: -P 2ybc	Cell parameters from 2969 reflections
*a* = 5.492 (1) Å	θ = 2.0–27.9°
*b* = 20.631 (4) Å	µ = 0.26 mm^−^^1^
*c* = 11.212 (2) Å	*T* = 113 K
β = 99.953 (6)°	Block, colourless
*V* = 1251.3 (4) Å^3^	0.18 × 0.12 × 0.10 mm
*Z* = 4	

### Data collection

**Table:**

Rigaku Saturn CCD area-detector diffractometer	2969 independent reflections
Radiation source: rotating anode	2499 reflections with *I* > 2σ(*I*)
multilayer	*R*_int_ = 0.036
Detector resolution: 14.63 pixels mm^-1^	θ_max_ = 27.9°, θ_min_ = 2.0°
ω and φ scans	*h* = −6→7
Absorption correction: multi-scan (*CrystalClear*; Rigaku, 2005)	*k* = −27→26
*T*_min_ = 0.954, *T*_max_ = 0.974	*l* = −14→14
9267 measured reflections	

### Refinement

**Table:**

Refinement on *F*^2^	Primary atom site location: structure-invariant direct methods
Least-squares matrix: full	Secondary atom site location: difference Fourier map
*R*[*F*^2^ > 2σ(*F*^2^)] = 0.040	Hydrogen site location: inferred from neighbouring sites
*wR*(*F*^2^) = 0.105	H atoms treated by a mixture of independent and constrained refinement
*S* = 1.05	*w* = 1/[σ^2^(*F*_o_^2^) + (0.058*P*)^2^ + 0.0584*P*] where *P* = (*F*_o_^2^ + 2*F*_c_^2^)/3
2969 reflections	(Δ/σ)_max_ = 0.001
169 parameters	Δρ_max_ = 0.22 e Å^−^^3^
0 restraints	Δρ_min_ = −0.46 e Å^−^^3^

### Special details

**Table:**

Geometry. All e.s.d.'s (except the e.s.d. in the dihedral angle between two l.s. planes) are estimated using the full covariance matrix. The cell e.s.d.'s are taken into account individually in the estimation of e.s.d.'s in distances, angles and torsion angles; correlations between e.s.d.'s in cell parameters are only used when they are defined by crystal symmetry. An approximate (isotropic) treatment of cell e.s.d.'s is used for estimating e.s.d.'s involving l.s. planes.
Refinement. Refinement of *F*^2^ against ALL reflections. The weighted *R*-factor *wR* and goodness of fit *S* are based on *F*^2^, conventional *R*-factors *R* are based on *F*, with *F* set to zero for negative *F*^2^. The threshold expression of *F*^2^ > σ(*F*^2^) is used only for calculating *R*-factors(gt) *etc*. and is not relevant to the choice of reflections for refinement. *R*-factors based on *F*^2^ are statistically about twice as large as those based on *F*, and *R*- factors based on ALL data will be even larger.

### Fractional atomic coordinates and isotropic or equivalent isotropic displacement parameters (Å^2^)

**Table:**

	*x*	*y*	*z*	*U*_iso_*/*U*_eq_	
S1	0.99680 (7)	0.594929 (18)	0.15089 (3)	0.02298 (13)	
O1	0.7267 (2)	0.54127 (5)	0.61880 (10)	0.0287 (3)	
O2	1.2319 (2)	0.57634 (7)	0.12187 (11)	0.0358 (3)	
O3	0.9462 (2)	0.66268 (6)	0.16584 (12)	0.0391 (3)	
N1	0.7215 (2)	0.63970 (6)	0.52476 (11)	0.0204 (3)	
N2	0.3756 (2)	0.68495 (6)	0.65521 (11)	0.0210 (3)	
N3	0.9639 (3)	0.55700 (7)	0.27108 (13)	0.0291 (3)	
H1N3	1.063 (4)	0.5283 (10)	0.2942 (19)	0.042 (6)*	
C1	0.6456 (3)	0.59733 (7)	0.60737 (14)	0.0212 (3)	
C2	0.6279 (3)	0.70370 (7)	0.50739 (14)	0.0211 (3)	
C3	0.4595 (3)	0.72539 (7)	0.57091 (13)	0.0222 (3)	
H3A	0.3970	0.7683	0.5587	0.027*	
C4	0.2052 (3)	0.69648 (8)	0.72848 (15)	0.0273 (4)	
H4A	0.1137	0.7353	0.7318	0.033*	
C5	0.1905 (3)	0.64143 (8)	0.79670 (15)	0.0305 (4)	
H5A	0.0872	0.6360	0.8557	0.037*	
C6	0.3529 (3)	0.59492 (7)	0.76456 (14)	0.0251 (4)	
H6A	0.3789	0.5524	0.7970	0.030*	
C7	0.4690 (3)	0.62255 (7)	0.67623 (14)	0.0214 (3)	
C8	0.9055 (3)	0.61459 (8)	0.45521 (14)	0.0230 (3)	
H8A	1.0242	0.5863	0.5079	0.028*	
H8B	0.9989	0.6512	0.4282	0.028*	
C9	0.7814 (3)	0.57612 (7)	0.34506 (14)	0.0240 (3)	
H9A	0.7018	0.5370	0.3721	0.029*	
H9B	0.6515	0.6029	0.2962	0.029*	
C10	0.7647 (3)	0.56615 (9)	0.03536 (15)	0.0304 (4)	
H10A	0.7739	0.5893	−0.0401	0.046*	
H10B	0.6024	0.5735	0.0580	0.046*	
H10C	0.7883	0.5196	0.0237	0.046*	
C11	0.7287 (3)	0.74663 (8)	0.41881 (14)	0.0267 (4)	
H11A	0.6390	0.7879	0.4110	0.040*	
H11B	0.7081	0.7252	0.3397	0.040*	
H11C	0.9047	0.7547	0.4481	0.040*	

### Atomic displacement parameters (Å^2^)

**Table:**

	*U*^11^	*U*^22^	*U*^33^	*U*^12^	*U*^13^	*U*^23^
S1	0.0228 (2)	0.0234 (2)	0.0228 (2)	−0.00198 (15)	0.00438 (15)	0.00159 (14)
O1	0.0331 (7)	0.0212 (6)	0.0301 (6)	0.0054 (5)	0.0009 (5)	0.0038 (4)
O2	0.0215 (6)	0.0569 (8)	0.0305 (7)	−0.0040 (6)	0.0083 (5)	−0.0009 (6)
O3	0.0553 (9)	0.0209 (6)	0.0401 (7)	−0.0029 (6)	0.0049 (6)	0.0035 (5)
N1	0.0229 (7)	0.0194 (6)	0.0188 (6)	0.0010 (5)	0.0036 (5)	0.0001 (5)
N2	0.0229 (7)	0.0194 (6)	0.0202 (6)	−0.0007 (5)	0.0024 (5)	−0.0013 (5)
N3	0.0360 (9)	0.0285 (7)	0.0253 (7)	0.0150 (7)	0.0128 (6)	0.0074 (6)
C1	0.0220 (8)	0.0197 (7)	0.0196 (7)	−0.0011 (6)	−0.0030 (6)	0.0012 (5)
C2	0.0240 (8)	0.0184 (7)	0.0194 (7)	−0.0029 (6)	−0.0004 (6)	0.0007 (5)
C3	0.0266 (8)	0.0167 (7)	0.0226 (7)	0.0002 (6)	0.0024 (6)	0.0014 (6)
C4	0.0254 (9)	0.0306 (8)	0.0272 (8)	−0.0007 (7)	0.0081 (7)	−0.0049 (7)
C5	0.0311 (10)	0.0349 (9)	0.0265 (9)	−0.0065 (7)	0.0074 (7)	−0.0029 (7)
C6	0.0301 (9)	0.0248 (8)	0.0196 (8)	−0.0065 (7)	0.0022 (6)	0.0007 (6)
C7	0.0237 (8)	0.0197 (7)	0.0192 (7)	−0.0016 (6)	−0.0009 (6)	0.0001 (6)
C8	0.0204 (8)	0.0245 (7)	0.0242 (8)	0.0014 (6)	0.0043 (6)	0.0004 (6)
C9	0.0253 (9)	0.0249 (8)	0.0232 (8)	0.0021 (6)	0.0080 (6)	−0.0010 (6)
C10	0.0217 (9)	0.0424 (10)	0.0272 (9)	−0.0040 (7)	0.0046 (7)	−0.0043 (7)
C11	0.0316 (9)	0.0227 (8)	0.0259 (8)	−0.0020 (7)	0.0053 (7)	0.0033 (6)

### Geometric parameters (Å, °)

**Table:**

S1—O2	1.4371 (12)	C4—C5	1.380 (2)
S1—O3	1.4405 (12)	C4—H4A	0.9500
S1—N3	1.5952 (14)	C5—C6	1.399 (2)
S1—C10	1.7563 (17)	C5—H5A	0.9500
O1—C1	1.2378 (17)	C6—C7	1.389 (2)
N1—C1	1.3890 (19)	C6—H6A	0.9500
N1—C2	1.4179 (19)	C8—C9	1.526 (2)
N1—C8	1.4731 (19)	C8—H8A	0.9900
N2—C4	1.3687 (19)	C8—H8B	0.9900
N2—C7	1.3907 (19)	C9—H9A	0.9900
N2—C3	1.3973 (19)	C9—H9B	0.9900
N3—C9	1.4616 (19)	C10—H10A	0.9800
N3—H1N3	0.82 (2)	C10—H10B	0.9800
C1—C7	1.438 (2)	C10—H10C	0.9800
C2—C3	1.338 (2)	C11—H11A	0.9800
C2—C11	1.506 (2)	C11—H11B	0.9800
C3—H3A	0.9500	C11—H11C	0.9800
			
O2—S1—O3	118.97 (8)	C7—C6—C5	107.08 (14)
O2—S1—N3	107.32 (8)	C7—C6—H6A	126.5
O3—S1—N3	108.98 (8)	C5—C6—H6A	126.5
O2—S1—C10	107.98 (8)	C6—C7—N2	107.42 (13)
O3—S1—C10	106.48 (8)	C6—C7—C1	132.03 (14)
N3—S1—C10	106.47 (8)	N2—C7—C1	120.49 (13)
C1—N1—C2	122.30 (13)	N1—C8—C9	111.09 (13)
C1—N1—C8	116.35 (12)	N1—C8—H8A	109.4
C2—N1—C8	121.35 (12)	C9—C8—H8A	109.4
C4—N2—C7	109.25 (13)	N1—C8—H8B	109.4
C4—N2—C3	129.93 (13)	C9—C8—H8B	109.4
C7—N2—C3	120.82 (13)	H8A—C8—H8B	108.0
C9—N3—S1	122.41 (12)	N3—C9—C8	110.19 (13)
C9—N3—H1N3	120.1 (14)	N3—C9—H9A	109.6
S1—N3—H1N3	117.2 (14)	C8—C9—H9A	109.6
O1—C1—N1	120.88 (14)	N3—C9—H9B	109.6
O1—C1—C7	123.13 (14)	C8—C9—H9B	109.6
N1—C1—C7	115.99 (13)	H9A—C9—H9B	108.1
C3—C2—N1	120.38 (13)	S1—C10—H10A	109.5
C3—C2—C11	121.40 (14)	S1—C10—H10B	109.5
N1—C2—C11	118.19 (13)	H10A—C10—H10B	109.5
C2—C3—N2	119.95 (14)	S1—C10—H10C	109.5
C2—C3—H3A	120.0	H10A—C10—H10C	109.5
N2—C3—H3A	120.0	H10B—C10—H10C	109.5
N2—C4—C5	107.54 (14)	C2—C11—H11A	109.5
N2—C4—H4A	126.2	C2—C11—H11B	109.5
C5—C4—H4A	126.2	H11A—C11—H11B	109.5
C4—C5—C6	108.71 (15)	C2—C11—H11C	109.5
C4—C5—H5A	125.6	H11A—C11—H11C	109.5
C6—C5—H5A	125.6	H11B—C11—H11C	109.5
			
O2—S1—N3—C9	163.37 (13)	N2—C4—C5—C6	−0.44 (19)
O3—S1—N3—C9	33.29 (16)	C4—C5—C6—C7	0.49 (19)
C10—S1—N3—C9	−81.20 (15)	C5—C6—C7—N2	−0.36 (17)
C2—N1—C1—O1	178.48 (14)	C5—C6—C7—C1	−177.60 (16)
C8—N1—C1—O1	−1.4 (2)	C4—N2—C7—C6	0.09 (17)
C2—N1—C1—C7	−1.1 (2)	C3—N2—C7—C6	179.43 (13)
C8—N1—C1—C7	179.04 (12)	C4—N2—C7—C1	177.72 (14)
C1—N1—C2—C3	−0.4 (2)	C3—N2—C7—C1	−2.9 (2)
C8—N1—C2—C3	179.51 (14)	O1—C1—C7—C6	0.1 (3)
C1—N1—C2—C11	177.87 (14)	N1—C1—C7—C6	179.63 (15)
C8—N1—C2—C11	−2.2 (2)	O1—C1—C7—N2	−176.85 (14)
N1—C2—C3—N2	0.2 (2)	N1—C1—C7—N2	2.7 (2)
C11—C2—C3—N2	−177.95 (14)	C1—N1—C8—C9	83.75 (16)
C4—N2—C3—C2	−179.39 (16)	C2—N1—C8—C9	−96.15 (16)
C7—N2—C3—C2	1.4 (2)	S1—N3—C9—C8	−100.44 (15)
C7—N2—C4—C5	0.21 (18)	N1—C8—C9—N3	174.55 (12)
C3—N2—C4—C5	−179.05 (15)		

### Hydrogen-bond geometry (Å, °)

**Table:**

*Cg*1 is the centroid of the N2/C4–C7 ring.

**Table:**

*D*—H···*A*	*D*—H	H···*A*	*D*···*A*	*D*—H···*A*
N3—H1N3···O1^i^	0.82 (2)	1.99 (2)	2.7923 (18)	168 (2)
C4—H4A···O3^ii^	0.95	2.36	3.258 (2)	157
C8—H8A···Cg1^iii^	0.99	2.96	3.5153 (19)	116

Symmetry codes: (i) −*x*+2, −*y*+1, −*z*+1; (ii) *x*−1, −*y*+3/2, *z*+1/2; (iii) *x*+1, *y*, *z*.
